# Generalizable disease detection using model ensemble on chest X-ray images

**DOI:** 10.1038/s41598-024-56171-6

**Published:** 2024-03-11

**Authors:** Maider Abad, Jordi Casas-Roma, Ferran Prados

**Affiliations:** 1 Universitat Oberta de Catalunya, e-Health Center, Barcelona, Spain; 2 Department of Computer Science, Universitat Autònoma de Barcelona (UAB), Bellaterra, Spain; 3 Computer Vision Center (CVC), Universitat Autònoma de Barcelona (UAB), Bellaterra, Spain; 4 Queen Square MS Centre, Department of Neuroinflammation, UCL Queen Square Institute of Neurology, Faculty of Brain Science, University College of London, London, WC1N 3BG UK; 5 Centre for Medical Image Computing (CMIC), Department of Medical Physics and Bioengineering, University College London, London, WC1V 6LJ UK

**Keywords:** Ensemble classifier, X-ray imaging, Transfer learning, Pre-trained models, Domain adaptation, Classification and taxonomy, Computational models, Image processing, Machine learning, Predictive medicine

## Abstract

In the realm of healthcare, the demand for swift and precise diagnostic tools has been steadily increasing. This study delves into a comprehensive performance analysis of three pre-trained convolutional neural network (CNN) architectures: ResNet50, DenseNet121, and Inception-ResNet-v2. To ensure the broad applicability of our approach, we curated a large-scale dataset comprising a diverse collection of chest X-ray images, that included both positive and negative cases of COVID-19. The models’ performance was evaluated using separate datasets for internal validation (from the same source as the training images) and external validation (from different sources). Our examination uncovered a significant drop in network efficacy, registering a 10.66% reduction for ResNet50, a 36.33% decline for DenseNet121, and a 19.55% decrease for Inception-ResNet-v2 in terms of accuracy. Best results were obtained with DenseNet121 achieving the highest accuracy at 96.71% in internal validation and Inception-ResNet-v2 attaining 76.70% accuracy in external validation. Furthermore, we introduced a model ensemble approach aimed at improving network performance when making inferences on images from diverse sources beyond their training data. The proposed method uses uncertainty-based weighting by calculating the entropy in order to assign appropriate weights to the outputs of each network. Our results showcase the effectiveness of the ensemble method in enhancing accuracy up to 97.38% for internal validation and 81.18% for external validation, while maintaining a balanced ability to detect both positive and negative cases.

## Introduction

Recent technological advancements in computer vision based on artificial intelligence (AI) applications have led to significant progress in X-ray image classification tasks^1–4^. Promising results point to its potential use as a supporting tool for clinicians; however, performance drops significantly when the models are deployed in real-world scenarios^5,6^. The challenges of achieving optimal results can be attributed to several factors. First, the difficulty generalizing the models implemented. Second, the limited availability of publicly accessible medical images with supervised pathological labels validated by qualified medical professionals presents a significant hurdle. Furthermore, the relatively small sample size of these data and the lack of agreement on labelling further compound the challenges of achieving robust, accurate results.

In situations where the available data is limited, transfer learning is a valuable and proven deep learning technique^7^. Transfer learning involves repurposing a model that was originally designed to tackle a different, but related problem or task. This approach has gained popularity in scenarios where there is a scarcity of annotated images and limited computational resources to train new models from scratch^8^. By leveraging pre-trained model architectures, transfer learning enables faster training processes with fewer input data, while improving overall model efficiency and generalization. This approach has significantly contributed to advancements in medical image artificial intelligence applications, as researchers and practitioners have successfully applied pre-trained models to enhance diagnostic capabilities across various medical imaging domains^9,10^.

COVID-19 diagnosis using medical imaging (i. e. chest X-rays) has accelerated thanks to computer vision techniques such as transfer learning. Apostolopoules and Mpesiana^11^ explored the effectiveness of convolutional neural network (CNN) models in detecting COVID-19, employing transfer learning techniques by utilizing pre-trained models from ImageNet to identify COVID-19 cases among various abnormalities. The MobileNetV2 model achieved 96.78% accuracy for COVID-19 positive (COVID-19+) and negative (COVID-19−) classification. A study by Chowdhury et al.^12^ trained and validated several models, including ResNet101, MobileNetV2, CheXNet, SqueezeNet, and DenseNet201, for both 2-class (COVID-19+ and COVID-19−) and 3-class (COVID-19+, COVID-19−, and Pneumonia) classification tasks. Among these models, DenseNet201 demonstrated the highest performance, achieving an accuracy of 99.7% for COVID-19 detection in the 2-class classification and an accuracy of 97.9% in the 3-class classification. Furthermore, a study by Manjural Ahsan et al.^13^ assessed the performance of six different pre-trained models for detecting COVID-19 from chest X-ray images and the results showed that VGG16 and MobileNetV2 gave the best outcomes, with up to 100% accuracy.

The excellent performance demonstrated in these studies can be attributed to the similarity between the training and the validation images in the data sets. However, when these top-performing models are extrapolated to different data sources, the lack of diversity in the training set and validation set images significantly decreases their performance. This means that current models lack the required robustness to be used in a clinical setting. Roberts et al.^14^ point out that while machine learning methods promise fast and accurate COVID-19 diagnosis and prognosis from chest X-ray images and computed tomography (CT), none of the models identified in their systematic review were of clinical utility due to methodological flaws and/or underlying biases. Additionally, Garcia Santa Cruz et al.^6^ emphasize the importance of rigorous evaluation of datasets used in AI models to ensure their validity and avoid biases in clinical practice. In their study, only nine out of over a hundred evaluated datasets met the criteria for proper assessment of the risk of bias, raising concerns about the suitability of models based on these datasets for clinical use. Furthermore, DeGrave et al.’s^5^ discussion of findings on the accuracy and robustness of AI systems in detecting COVID-19 from chest X-rays reveals that current deep learning systems rely on confounding factors rather than medical pathology, leading to potential failures when applied in new hospitals. The study also highlights that the data collection approach allows AI to learn spurious shortcuts, which is a widespread issue in AI-driven medical imaging.

These findings indicate that current models exhibit limitations and biases, highlighting that certain challenges remain unresolved before achieving successful clinical application. The main problem lies in the data utilized for training the model, as it is crucial to avoid bias by incorporating data from different sources than those used for training when assessing the model’s ability to generalize. This ensures a more robust evaluation and reduces the risk of the model being overly specialized to a particular dataset.

Another approach that enhances the model’s generalization capability is employing model ensemble techniques. This involves combining multiple models, each trained on different subsets of data or using different algorithms, to create a more powerful and diverse predictive system. Leveraging the collective knowledge and strengths of these models improves both performance and adaptability to unseen data. Ensembling can help mitigate individual model limitations, thus increasing overall accuracy and reliability.

In recent years, several studies have emerged focusing on the application of ensemble modeling for COVID-19 detection. Chowdhury et al.^15^ employed both hard-voting (majority voting) and soft-voting (averaging) techniques with EfficientNet family networks to classify COVID-19, normal, or pneumonia. During their validation, they utilized 1579 images, which included 100 COVID-19+ cases and originated from the same sources as the training data, leading to an overall accuracy of 96.07%.

Das et al.^16^ applied averaging for ensemble modeling, incorporating DenseNet201, ResNet50V2, and Inceptionv3 for binary classification. The internal validation was performed using images from the same sources as the training data, specifically the used 117 images, including 57 COVID-19+ cases, achieving an accuracy of 91.60%.

Meanwhile, Paul et al.^17^ proposed an ensemble method based on an inverted bell curve weighted ensemble, employing Densenet-161, ResNet18, and VGG-16 networks. Their binary and 3-class classifications were conducted on an internal validation dataset comprising 1214 images, including 683 COVID-19+ cases, with an outstanding accuracy of 99.84%.

It’s worth noting that only a limited number of authors have undertaken external validations to assess the robustness of their models. Deb et al.^18^ implemented feature concatenation on VGGNet, GoogleNet, DenseNet, and NASNet networks for binary and three-class classification (COVID-19, normal, and community-acquired pneumonia). Their validation included an internal dataset of 1626 images (136 COVID-19+) and an external dataset of 92 images (29 COVID-19+) sourced from a single origin. The outcomes exhibited an accuracy of 98.58% for internal binary classification validation and 95.65% for external validation. Wehbe et al.^19^ advocated for the usage of a weighted average in ensemble modeling with DenseNet-12, ResNet-50, InceptionV3, Inception-ResNetV2, Xception, and EfficientNet-B2 networks. The binary classification was conducted with external validation on 2214 images (1192 COVID-19+) from a singular source, resulting in an accuracy of 82.00%.

Other researchers proposed a strategy employing the same architectural framework with multiple instances for ensemble modeling. Kuo et al.^20^ applied an equally weighted ensemble to four instances of RadGenX. External validation for binary classification was executed on 5894 images (2747 COVID-19+), yielding an AUC of 79.00%. Similarly, Miyazaki et al.^21^ employed an averaging ensemble approach on five instances of EfficientNet for binary classification. Validation on an external dataset of 180 images (60 COVID-19+) from a singular source produced an accuracy of 73.30%. Many studies in the current literature concentrate on conducting validations using images from the same sources as those used in training. Only a few have implemented external validation, but they typically rely on a single external data source. Furthermore, the prevalent ensemble methods often rely on techniques like averaging or weighted ensemble.

Given these challenges, our paper introduces a novel ensemble methodology grounded in entropy to weigh models’ outputs, contributing to advancements in medical image classification and fortifying methodologies within the healthcare sector. The primary contributions are as follows:

- Creating a robust COVID-19 detection model through transfer learning on pre-trained CNNs from ImageNet.
- Assessing the model’s generalization on diverse internal and external validation sets, validating its ability to generalize across different datasets.
- Introducing a novel entropy technique to weigh model outputs, striving for a more accurate overall result when combining the models.

We work under the assumption that training with a comprehensive dataset covering all possible medical images worldwide is impractical. Instead, we acknowledge that models available for use have been trained on datasets that differ from those specific to individual hospitals. The core idea is that combining various models can offer an enhanced solution, addressing the variability in image datasets encountered across different healthcare facilities.

This research not only serves as a proof of concept for streamlining the medical image classification process but also contributes to the advancement and fortification of these methodologies within the healthcare sector.

## Materials and methods

The following section outlines the datasets and methods used in this research.

### Dataset

The datasets used:

- The COVIDx CXR-3 dataset^22^ comprises 30,386 X-ray images, including 16,194 positive COVID-19 cases and 14,192 negative cases. The COVIDx CXR-3 dataset was compiled from 8 different public data sources. No metadata is associated with the images in this database.
- The COVIDGR dataset^23^ is a curated collection of chest X-ray images annotated with findings related to COVID-19, and contains 426 positive cases and 426 negative cases. Positive cases have accompanying metadata indicating the severity of the illness on a scale ranging from severe to moderate, mild, and normal-PCR+.
- The Labeled Optical Coherence Tomography (OCT) and Chest X-Ray Images for Classification dataset^24^ is a publicly available collection of chest X-ray and OCT images. The chest X-rays were obtained from the University of California San Diego and are labeled as either “normal” or “pneumonia” to indicate the presence or absence of the disease. The total dataset comprises 1583 normal and 4273 pneumonia images. For this study, which aims to differentiate between COVID-19+ and COVID-19− images, only the images labeled “normal” were used. No metadata is associated with the images in this database.

Table 1 provides information on the data sources and database division for the training, internal validation, and external validation groups. These groups comprised 13,534 COVID-19+ and 12,513 COVID-19− images for training, 1294 COVID-19+ and 1382 COVID-19− images for internal validation, and 1792 COVID-19+ and 2306 COVID-19− images for external validation. The absence of metadata underscores the importance of carefully selecting an external validation dataset, ensuring that the source of the images differs from those used in internal validation or training. It is crucial to highlight that this divergence involves images originating from different hospitals, each utilizing various imaging acquisition machines. Additionally, ensuring the proper calibration of both positive and negative cases has been implemented.

The external validation dataset comprised images from a number of sources, one of which was COVIDGR^23^. From this source, a total of 426 images of positive cases were utilized, with severity data available on the Severe-Moderate-Mild-Normal-PCR+ scale, which includes 79 Severe cases, 171 Moderate cases, 100 Mild cases, and 76 Normal-PCR+ cases.

**Table 1:** Summary of the datasets used in the research.

Database name	Data source	Number of images per class	Train, internal or external validation
COVIDx CXR-3^22^	Covid-chestxray-dataset^25^ Figure 1 COVID-19 Chest X-ray Dataset Initiative^26^ Actualmed COVID-19 Chest X-ray Dataset Initiative^27^ Italian Society of Medical and Interventional Radiology (SIRM)^28^ RSNA Pneumonia Detection Challenge^29^ RSNA International COVID-19 Open Radiology Database (RICORD)^30^ BIMCV-COVID19+: a large annotated dataset of RX and CT images of COVID19 patients^31^ Stony Brook University COVID-19 Positive Cases (COVID-19-NY-SBU)^32^	270 COVID-19+, 297 COVID-19− 24 COVID-19+ 25 COVID-19+, 107 COVID-19− 943 COVID-19+ 13,788 COVID-19− 1096 COVID-19+ 200 COVID-19+ 13,636 COVID-19+	External validation Train-internal validation Train-internal validation Train-internal validation Train-internal validation External validation Train-internal validation Train-internal validation
COVIDGR^23^	Hospital Universitario Clínico San Cecilio, Granada, Spain	426 COVID-19+, 426 COVID−	External validation
Labeled optical coherence tomography (OCT) and chest X-ray images for classification^24^	The University of California, San Diego, CA	1583 COVID−	External validation

### Study design

Five main steps were followed:

1. All the images by source and category (positive and negative) were collected and grouped.
2. The dataset was divided into three sets: training, internal validation, and external validation. Without metadata for in-depth analysis, the preparation of the dataset before feeding it to the neural network has been based on ensuring balanced classification and avoiding overlap between image sources in the training and internal validation sets compared to those in the external validation set. The inclusion of images of the same subject in the same set was consistently maintained. Consequently, due to these constraints, the percentages for each class may slightly deviate from the intended values of 75% for training, 10% for internal validation, and 15% for external validation.
3. Transfer learning was applied to three pre-trained networks using ImageNet.
4. The models’ performance was assessed using both internal and external datasets. Internal validation refers to using images from the same source as the training images, while external validation involved using images from different sources.
5. The outputs of all the models were combined to obtain a joint solution.

**Figure 1:**
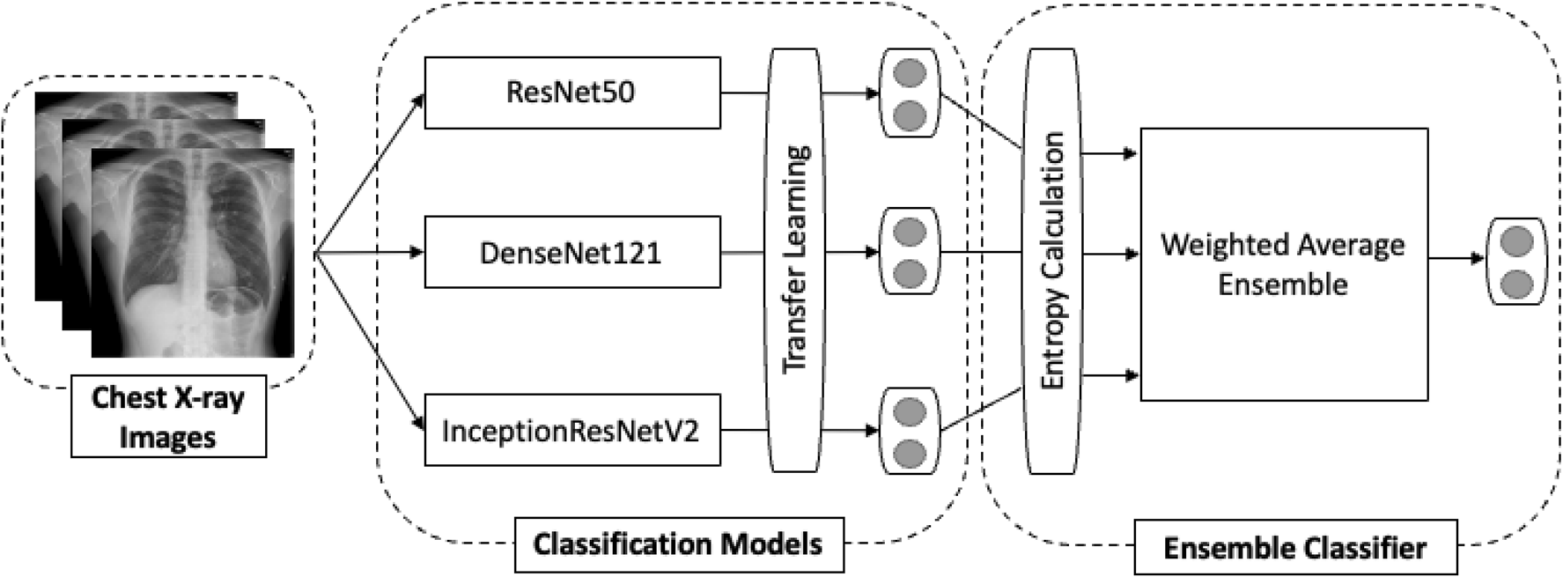
Proposed model ensemble architecture for COVID-19 detection.

Figure 1 shows the project workflow.

### Model training

The study used three pre-trained CNN architectures, namely Inception-ResNet-v2 (IRV2)^33^, ResNet50^34^, and DenseNet121^35^, all of which were originally trained on the ImageNet dataset. The selection of the networks was driven not by their distinctiveness but by their widespread use in image classification^19,36,37^. Opting for these architectures, instead of more sophisticated alternatives, was intended to streamline reproducibility and enhance experiment understanding, ultimately emphasizing the inherent difficulty of generalizing the models. Importantly, this pipeline proposal remains flexible and does not preclude the utilization of other pre-trained models. To apply transfer learning, all layers in the CNNs were frozen, and a classifier was added to the top of each network.

All input images were in either png, jpg, or jpeg format and were preprocessed by normalizing their pixel values to between 0 and 1. The images were also resized to the standard $256 \times 256 \times 3$ pixels using bilinear interpolation, with the same image repeated in all colour channels. This resizing approach calculates pixel values in the resized image through linear interpolation, referencing surrounding pixel values from the original image. The choice of this image size was selected to strike a balance between model accuracy and computational efficiency^38,39^.

To construct the classifier, a series of layers were added to the pre-trained CNN architectures. These included a global average pooling layer, three fully connected (FC) layers with 128 (FC1-Dense), 64 (FC2-Dense), and 16 nodes (FC3-Dense), respectively, and ReLU activation. A dropout layer was added after each fully connected layer with a rate of 0.3 to prevent overfitting, and the 2-node dense output layer was activated by the softmax function.

The global average pooling layer computes the average of each feature map in the final convolutional layer, giving a fixed-length vector for each image. This vector was then fed into the subsequent layers. The fully connected layers performed a series of linear transformations on the input data and the ReLU activation function was applied to introduce non-linearity. The dropout layer randomly eliminated some nodes to prevent overfitting. Finally, the softmax function was applied to the output dense layer to predict probabilities for each class. These layers worked together to transform the CNN output into a probability distribution over the two classes.

**Figure 2:**
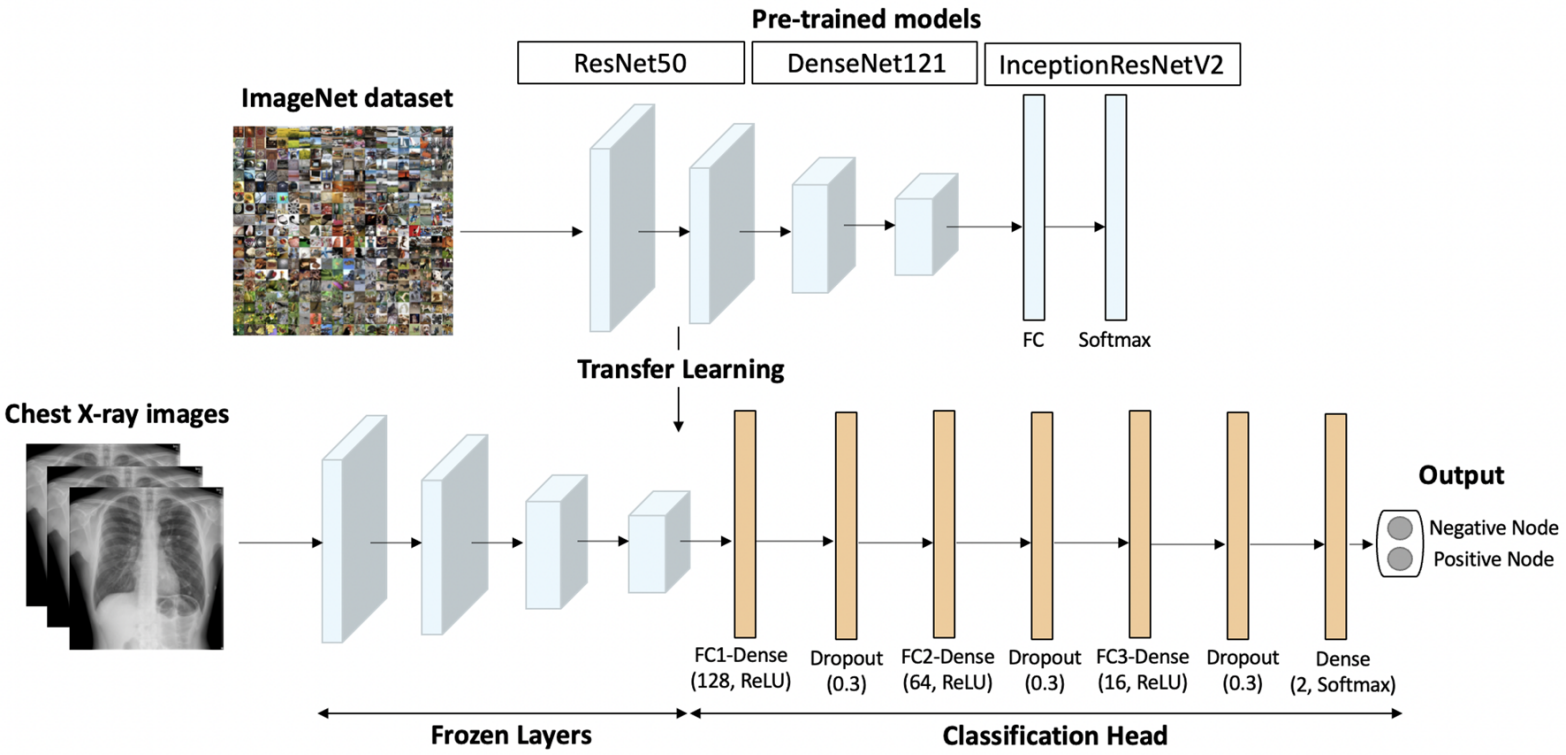
Flowchart of proposed transfer learning model.

The models were trained for 50 epochs, with a batch size of 128 and an Adam optimizer with a learning rate of $10^{-4}$. To prevent overfitting during training, the regularization technique employed was early stopping, where training was stopped on the criterion of a significant increase in loss. The CNN architectures and associated layers were selected and optimized to achieve accurate, efficient classification of images into two classes. Figure 2 illustrates the transfer learning architecture.

### Model ensemble

In this study, we applied uncertainty-based weighting and entropy calculation to weight the outputs of different networks. Uncertainty-based weighting is a technique that aims to improve the accuracy of ensemble models by assigning different weights to each model output based on its level of uncertainty^40–42^. In this case, entropy is used as a measure of uncertainty, with higher entropy indicating greater uncertainty in the model’s predictions.

This weighting technique involves calculating the entropy for each model’s prediction for a given input data point *x*. The entropy $H(O_i(x))$ of each model *i* is calculated using Eq. (1),

1 $$\begin{aligned} H(O_i(x))= - \sum _{j=1}^c p_i(j) \times \log _2(p_i(j)) \end{aligned}$$

where $p_i(j)$ is the predicted probability of class *j* for model *i*, and *c* is the total number of classes. In our case, $c=2$ as the variable *j* can take on two values: 1 or 2 (COVID-19− or COVID-19+). When $p_i(j=1)$ means that the predicted probability that the data point *x* belongs to the COVID-19− class using the model *i*. Conversely, $p_i(j=2)$ represents the probability that image *x* belongs to the COVID-19+ class using the model *i*.

The negative exponential of the entropies for each model is then summed up to obtain the denominator for the weight calculation using Eq. (2),

2 $$\begin{aligned} \sum _{k=1}^{m} e^{-H(O_k(x))} \end{aligned}$$

where *m* is the total number of models, as three different models are used: ResNet50, DenseNet121 and IRV2, $m=3$.

The weight *w* for each model *i* is calculated using Eq. (3), in which the negative exponential of the entropy of the models (Eq. 1) is divided by the sum of all negative exponentials of the entropies (Eq. 2).

3 $$\begin{aligned} w_i = \frac{e^{-H(O_i(x))}}{\sum _{k=1}^{m} e^{-H(O_k(x))}}, \text { where } i \in [1, \ldots , m] \end{aligned}$$

Finally, the total weighted output *O*(*x*) for each class *j* and model *i* is calculated using Eq. (4),

4 $$\begin{aligned} O(x) = \sum _{i=1}^{m} w_i \times p_i(j) \end{aligned}$$

where $w_i$ is the weighting factor for the model *i* and $p_i(j)$ is the predicted probability of class *j* for model *i*.

Using uncertainty-based weighting with entropy calculation, we can exploit the strengths of different models, thus improving the overall performance of the ensemble model. This technique also helps reduce the impact of outliers or poorly performing models, as their weights are lower due to their higher level of uncertainty. Furthermore, the use of entropy provides a mathematically rigorous method for measuring uncertainty, which can be particularly useful in complex or high-dimensional data.

### Evaluation metrics

Various metrics were employed to assess the model’s performance. These included accuracy, sensitivity/recall, specificity, precision, F1 score, and area under the curve (AUC). These measures were labelled thus: true positive (TP); true negative (TN); False positive (FP); and false negative (FN). TP refers to a subject with COVID-19 who tests positive; TN denotes a subject who does not have the disease and tests negative. FP corresponds to a subject who does not have COVID-19 but tests positive, and FN denotes a subject who has COVID-19 but tests negative.

Sensitivity, as shown in Eq. (5), is particularly noteworthy. A classifier with 100% sensitivity correctly identifies all positive cases with the disease, which is crucial for detecting severe illnesses.

5 $$\begin{aligned} Sensitivity = Recall = \frac{TP}{TP+FN} \end{aligned}$$

In addition to sensitivity, the study also assessed the specificity of the model, which measures the proportion of true negatives the model correctly identifies. Specificity is calculated using Eq. (6),

6 $$\begin{aligned} Specificity = \frac{TN}{FP+TN} \end{aligned}$$

The accuracy of the model was also evaluated. Accuracy is a widely used parameter in evaluating classifier performance and provides an overall assessment of the model’s effectiveness. It is defined using Eq. (7),

7 $$\begin{aligned} Accuracy = \frac{TP+TN}{TP+TN+FP+FN} \end{aligned}$$

Precision and the F1 score, as indicated in Eqs. (8) and (9), were also calculated to assess the model’s performance. Precision indicates how well the model correctly identifies positive cases and is represented as,

8 $$\begin{aligned} Precision = \frac{TP}{TP+FP} \end{aligned}$$

The F1 score is a statistical measure that considers the model’s precision and recall in its calculation and yields a value between 0 and 1.

9 $$\begin{aligned} F1 = 2 \times \frac{Precision \times Recall}{Precision+Recall} = \frac{2TP}{2TP+FP+FN} \end{aligned}$$

For all metrics, 95% confidence intervals (CI) have been calculated. Additionally, a two-tailed t-test has been conducted to compare the performance of the proposed ensemble method with the rest of the classifiers. The Null Hypothesis (H0) suggests that there is no significant difference between the means of the two models. A p value below 0.05 was considered statistically significant; therefore, if the p value is less than 0.05, there would be sufficient evidence to reject the null hypothesis.

## Results and discussion

### Performance on internal validation dataset

**Figure 3:**
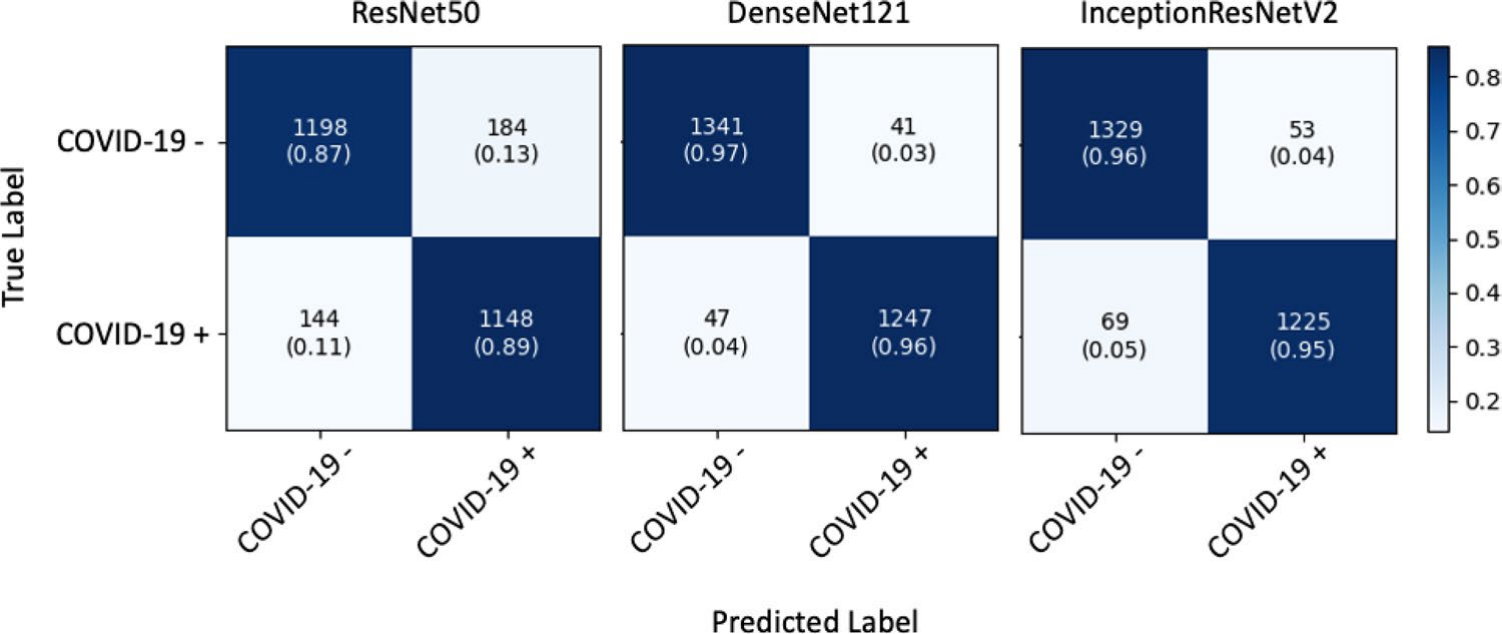
Confusion matrix of transfer learning models on the internal dataset.

In the first experiment, we used the internal validation set to evaluate the performance of the three networks alone. The confusion matrix obtained for each network is shown in Fig. 3. A more detailed analysis of the corresponding data is provided in Table 2. The best results were obtained using DenseNet121, achieving an accuracy of 96.71%, precision of 96.82%, sensitivity of 96.37%, specificity of 97.03%, F1 score of 96.59% and AUC of 96.70%.

**Table 2:** Comparing internal validation results for the proposed ensemble model against transfer learning models. 95% CI is represented as [lower bound–upper bound].

Model	Accuracy (%)	Precision (%)	Sensitivity (%)	F1 score (%)	Specificity (%)	AUC (%)
ResNet50	87.73*	86.19*	88.85*	87.50*	86.69*	88.77*
	[86.02–89.46]	[84.00–88.57]	[87.26–90.48]	[86.02–89.21]	[84.16–89.21]	[86.07–89.48]
DenseNet121	96.71	96.82*	96.37	96.59	97.03*	96.70
	[96.04–97.39]	[96.04–97.61]	[95.26–97.47]	[96.03–97.39]	[96.28–97.78]	[96.02–97.38]
IRV2	95.44*	95.85*	94.67*	95.26*	96.16*	95.42*
	[94.53–96.36]	[94.55–97.23]	[93.21–96.13]	[94.52–96.36]	[94.88–97.46]	[94.50–96.34]
Proposed assembling	97.38	98.12	96.45	97.38	98.26	97.35
	**[96.76–98.01]**	**[97.48–98.77]**	**[95.25–97.64]**	**[96.75–98.01]**	**[97.66- 98.87]**	**[96.71–98.00]**

Significant values are in [bold].

^∗^A statistically significant difference (p < 0.05) when comparing against the proposed assembling method.

### Performance on external validation dataset

**Figure 4:**
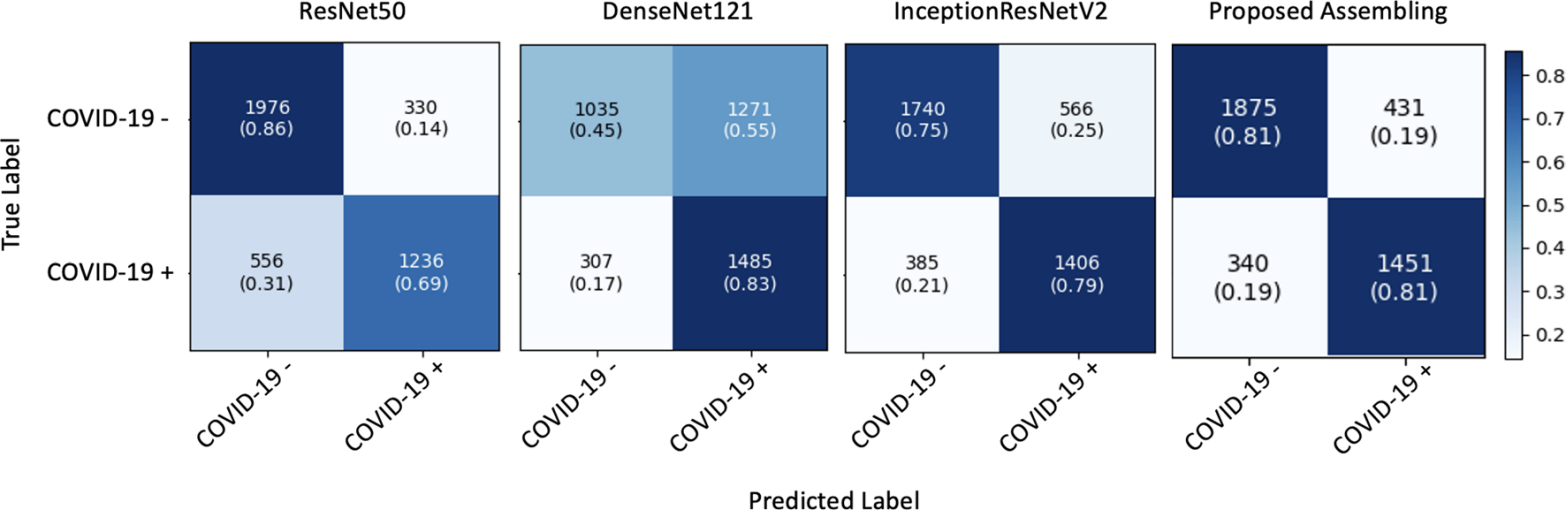
Confusion matrix of transfer learning models on the external dataset.

In the second experiment, we used an external dataset comprising images taken from different sources to those used for training or internal validation. The confusion matrix obtained for each network is shown in Fig. 4. A more detailed analysis of the corresponding data is provided in Table 3.

**Table 3:** Comparison of performance of external validation for proposed assembling model and transfer learning models.

Model	Accuracy (%)	Precision (%)	Sensitivity (%)	F1 score (%)	Specificity (%)	AUC (%)
ResNet50	78.38*	78.93*	68.97*	78.15*	85.69*	77.33*
	[76.93–79.82]	**[77.15–80.72]**	[66.51–71.44]	[76.67–79.63]	**[84.36–87.02]**	[75.80–78.87]
DenseNet121	61.49*	53.93*	82.91*	60.48*	44.89*	63.88*
	[59.31–63.71]	[52.23–55.63]	**[80.77–85.06]**	[58.07–62.87]	[41.86–47.92]	[61.78–66.02]
IRV2	76.79*	71.42*	78.50*	76.87*	75.46*	76.96*
	[74.69–78.89]	[68.75–74.10]	[76.08–80.83]	[74.79–78.95]	[72.78–78.21]	[74.91–79.04]
Proposed assembling	**81.16**	77.11	80.97	**81.20**	81.31	**81.14**
	**[78.99–83.33]**	[74.69–79.53]	[78.21–83.73]	**[79.04–83.37]**	[79.26–83.36]	**[78.99–83.33]**

95% CI is represented as [lower bound-upper bound].

Significant values are in [bold].

^∗^A statistically significant difference (p < 0.05) when comparing against the proposed assembling method.

The models’ performance shows a notable decline in this scenario, with ResNet50 yielding the best results at accuracy at 78.38%, precision at 78.93%, specificity at 85.69%, F1 score of 78.15% and AUC of 77.33%. In terms of sensitivity, DenseNet121 achieved the best results at 82.91%.

Furthermore, our research focused on examining the effectiveness of severity-based COVID-19 detection by analyzing images from the COVIDGR^23^ dataset. The TP percentages for each class are presented in Table 4. Overall, the models demonstrated a higher accuracy in correctly identifying more severe cases; but faced challenges in accurately classifying milder cases.

**Table 4:** Analysis of true positive (TP) percentages in COVID-19 detection based on severity levels using the COVIDGR dataset.

	Severe (%)	Moderate (%)	Mild (%)	Normal-PCR+ (%)
ResNet50	44.31	50.29	40.00	31.5%
DenseNet121	77.22	**58.48**	**50.00**	**46.05**
IRV2	**81.01**	54.97	35.00	26.32
Proposed assembling	75.32	57.31	40.00	27.37

Significant values are in [bold].

On analyzing the origin of the images, 70.44% of the images classified as FN were found to belong to the COVIDGR database^23^, and 53.59% of the images classified as FP belonged to the covid-chestxray-dataset^25^ (one of the 8 data sources making up the COVIDx CXR-3 dataset). This source contains samples from patients who have tested positive or are suspected of having COVID-19 and samples from patients with other viral and bacterial pneumonia such as MERS, SARS or ARDS. This may have led to some images with different pathologies being interpreted as positive cases.

### Model ensemble

The confusion matrix of the assembling model and the comparison between each individual model’s performance and the ensemble are shown in Fig. 4 and Table 3.

The combination of models demonstrates improved classification for cases of both COVID-19+ and COVID-19−. Regarding internal validation, the model assembly enhances the results obtained by individual networks, achieving an accuracy of 97.38% and AUC of 97.35, as shown in Table 2. During external validation, certain aspects, such as sensitivity and specificity, performed better in other models, as shown in Table 3. However, these models exhibited weaknesses in other areas; for instance, ResNet50 achieved a specificity of 85.69% (p < 0.05), but its sensitivity was only 68.97% (p < 0.05), whereas DenseNet121 attained a sensitivity of 82.91% (p < 0.05), but its specificity dropped to 44.89% (p < 0.05). Therefore, the proposed model assembly in this study achieved a balanced solution, yielding a sensitivity of 80.97% and a specificity of 81.31%. These values represent the highest overall accuracy of 81.16% and AUC of 81.14%.

Regarding the severity study, the results in Table 4 indicate that both individual models and the model ensemble have higher detection rates for cases labeled as severe than cases classified as mild or normal-PCR+.

### Benchmarking ensembling models

To assess the robustness of our ensemble model, we first conducted a performance comparison with other commonly used ensemble models using our external validation dataset. Specifically, we chose soft-voting methods that involve averaging and weighted averaging. For weighted averaging, we adopted an approach where weights are generated randomly using a Dirichlet distribution^43^. Additionally, we considered hard-voting methods based on majority voting. The findings in Table  5 reveal that, for the external validation dataset, the approach proposed in this article demonstrates superior overall performance compared to the other three methods. The only exception arises in the sensitivity measurement between averaging soft voting and the proposed ensemble method, where no statistically significant difference has been observed (p > 0.05).

**Table 5:** Comparing performance of external validation for the proposed ensemble model against other ensemble methods.

Category	Method	Accuracy (%)	Precision (%)	Sensitivity (%)	F1 score (%)	Specificity (%)	AUC (%)
Soft-voting	Averaging	80.64*	76.21*	**81.02**	80.69*	80.36*	80.64*
		[78.59–82.70]	[74.04–78.38]	**[78.14–83.89]**	[78.65–82.74]	[78.56–82.16]	[78.56–82.81]
	Weighted averaging	78.24*	72.93*	80.08*	78.35*	76.89*	78.48*
		[76.07–80.50]	[70.62–75.24]	[76.94–83.22]	[76.15–80.56]	[74.73–79.05]	[76.20–80.76]
Hard-voting	Majority voting	79.64*	74.89*	80.46*	79.70*	79.01*	79.73*
		[77.51–81.78]	[72.45–77.33]	[77.70–83.21]	[77.58–81.83]	[76.79–81.24]	[77.57–81.90]
Proposed assembling		**81.16**	**77.11**	80.97	**81.20**	**81.31**	**81.14**
		**[78.99–83.33]**	**[74.69–79.53]**	[78.21–83.73]	**[79.04–83.37]**	**[79.26–83.36]**	**[78.99–83.33]**

95% CI is represented as [lower bound–upper bound].

Significant values are in [bold].

^∗^ denotes a statistically significant difference (p < 0.05) when comparing against the proposed assembling method.

### Comparison to the state-of-the-art results

This article has conducted a comparative analysis contrasting our proposed ensemble approach with various state-of-the-art ensemble models applied to COVID-19 classification. Table  6 presents the results of these studies along with the methodologies employed and the type of validation performed.

Among the 14 studies scrutinized in the ensemble methods comparison for COVID-19 detection within the state of the art, merely 4 conducted external validation using images from sources distinct from those used in internal training and validation. Of these 4 studies, only 2 utilized more than one network and demonstrated results surpassing those of our model.

In the first case, Deb et al.^18^ implemented feature concatenation for four different models and assessed them using an external database comprising 92 images, with 29 belonging to the COVID-19+ class. They achieved an accuracy of 93.48% for the classification of 3 classes and 95.65% for binary classification. The number of images used in this study for external validation is considerably limited compared to our study, which involved a more extensive dataset comprising 4098 images, including 1792 COVID-19+ cases from four distinct sources.

In the second case, Wehbe et al.^19^ employed a weighted average ensemble with 6 different models for binary classification. They evaluated these models on an external database containing 2214 images, of which 1192 were COVID-19+ and originated from a single source. The results exhibited an accuracy gain of 0.84% compared to our method, along with an increase of 6.86% in AUC, 11.69% in specificity and a decrease of 9.97% in sensitivity. In our comparison with commonly used ensemble models, we applied the same methodology as presented in this article when comparing weighted averaging. Notably, in our case, the performance of the proposed ensemble method remains statistically significantly superior to the weighted averaging approach as seen in Table  5.

**Table 6:** Comparing state-of-the-art results obtained from published ensemble methods for COVID-19 detection.

Article	Method	Model	# Classes	Validation type (external/internal)	Validation sample	Accuracy (%)	Precision (%)	Sensitivity (%)	F1 (%)	Specificity (%)	AUC (%)
Hussain et al.^44^	Bagging	EfficientNet-B0, VGG-16, and DenseNet201	3	Internal	1131 (377 COVID-19+)	97.00	96.00	95.00	97.00	–	–
Chowdhury et al.^15^	Hard ensemble and Soft ensemble	EfficientNet family networks	3	Internal	1579 (100 COVID-19+)	96.07 (hard ensemble) 96.07 (soft ensemble)	94.17 (hard ensemble) 92.59 (soft ensemble)	97.00 (hard ensemble) 100.00 (soft ensemble)	95.57 (hard ensemble) 96.15 (soft ensemble)	–	–
Tang et al.^45^	Weighted averaging	COVID-NET-M1 toM6	3	Internal	1579 (100 COVID-19+)	94.60	94.10	96.00	–	–	–
Das et al.^16^	Averaging	DenseNet201, ResNet50V2,and Inceptionv3	2	Internal	117 (57 COVID-19+)	91.60	–	95.09	91.71	–	91.71
Paul et al.^17^	Inverted bell curve weighted ensemble	Densenet-161, ResNet18 and VGG-16	3	Internal	582 (46COVID-19+)	99.66	–	–	99.75	–	99.99
			2		1214 (683 COVID-19+)	99.84	–	–	99.81	–	99.99
Breve et al.^46^	Averaging	21 different CNN architectures; the best model was obtained by ensembling five instances of DenseNet169	2	Internal	400 (200 COVID-19+)	99.25 (best model)	100.00 (best model)	98.50 (best model)	99.24 (best model)	–	–
Balasubramaniam et al.^47^	Averaging	Support Vector Machine (SVM), CNN, Optimized Neural Network (NN), and Random Forest (RF)	2	Internal	3100 (100 COVID-19+)	96.64	95.24	93.66	–	92.57	–
Pramanik et al.^48^	TOPSIS	3 customised CNNs	3	Internal	1229 (185 COVID-19+) (Dataset 1)	98.78	98.47	98.26	98.37	–	–
					3030 (723 COVID-19+) (Dataset 2)	98.61	97.84	97.85	97.85	–	–
Deb et al.^18^	Feature concatenation	VGGNet, GoogleNet, DenseNet, and NASNet	3	Internal	1626 (136 COVID-19+)	88.92	98.00	62.00	75.00	–	–
				External	92 (29 COVID-19+)	93.48	100.00	86.00	93.00	–	–
			2	Internal	1626 (136 COVID-19+)	98.58	87.41	97.05	91.97	–	–
				External	92 (29 COVID-19+)	95.65	88.31	95.05	91.56	–	–
Eshraghi et al.^49^	Weighted sum	MobileViT and MobileNetV3	2	Internal	400 (200 COVID-19+)	97.75	97.04	98.05	97.78	–	–
Wehbe et al.^19^	Weighted average	DenseNet-12, ResNet-50,InceptionV3, Inception-ResNetV2, Xception, and EfficientNet-B2	2	External	2214 (1192 COVID-19+)	82.00	–	71.00	–	93.00	88.00
Nishio et al.^50^	Majority voting	Five instances of EfficientNet	3	Internal	300 (100 COVID-19+) (Dataset 1)	–	–	–	–	–	99.34
					300 (100 COVID-19+) (Dataset 2)	–	–	–	–	–	98.56
					150 (50 COVID-19+) (Dataset 3)	86.67	86.54	90.00	88.24	–	97.52
Kuo et al.^20^	Equally weighted ensemble	Four instances of RadGenX	2	External	5894 (2747 COVID-19+)	–	–	79.10	–	60.50	79.00
Miyazaki et al.^21^	Averaging	Five instances of EfficientNet	3	External	180 (60 COVID-19+)	73.30	–	66.70	–	78.30	78.60
Our model	Entropy	ResNet50, DenseNet121 and IRV2	2	Internal	2676 (1294 COVID-19+)	97.38	98.12	96.45	97.38	98.26	97.35
				External	4098 (1792 COVID-19+)	81.16	77.11	80.97	81.20	81.31	81.14

### Discussion

This study compared the performance of three pre-trained neural networks on an internal validation and an external validation dataset. Results showed that the models performed exceptionally well on the internal validation dataset, where the images are from the same source as the training dataset. DenseNet121 achieved the highest AUC (96.70%) on the internal validation dataset.

However, when we tested the same models on the external validation dataset, which contains images from a different source, performance dropped significantly. ResNet50 attained the highest AUC on the external validation dataset, reaching 77.33%.

Combining the output of the models has demonstrated improved classification performance, with AUC for the internal validation dataset rising to 97.35%, and external validation rising to 81.14%. This study used 3 models as proof of concept to demonstrate the contribution of network ensemble. However, this methodology can be extrapolated to a larger number of networks to achieve more robust results.

Additionally, the results of the t-test, which compares the performance of the ensemble model against each individual network, indicate that, in the case of internal validation, the ensemble outperforms the IRV2 and ResNet50 networks statistically. For DenseNet121, no significant differences are observed, except in precision and specificity values, where our ensemble shows better performance with p < 0.05. Regarding external validation, the proposed ensemble has demonstrated significantly higher accuracy, F1 score, and AUC compared to each individual network.

Regarding the severity analysis, the results in Table 4 reveal that the proposed ensemble of models is not the most suitable for detecting COVID-19+ cases for the severity labels specified. Considering that the number of images in the dataset containing severity metadata is relatively small, this may potentially limit the generalizability of the findings. Furthermore, the limited sample size may affect changes in percentages within the same categories, and therefore its impact. Nevertheless, it is worth noting that there is a noticeable tendency to classify severe cases with greater accuracy.

To highlight the robustness of our ensemble methodology, a performance comparison was conducted with commonly used methods in the literature, such as soft-voting and hard-voting. The results demonstrated that our proposed ensemble achieves the best outcomes. Thus, by combining the strengths and mitigating the weaknesses of individual models, a global model was developed that significantly enhances performance. This research not only serves as a proof of concept for streamlining the medical image classification process but also contributes to the advancement and fortification of these methodologies within the healthcare sector. Furthermore, the exploration of combining results from networks trained under diverse circumstances underscores the potential to improve overall performance, particularly when confronted with data unfamiliar to any of the individual networks.

On analyzing the results of the external validation dataset, we noted two factors that may influence network performance.

First, we found the COVIDGR^23^ source highly effective for detecting severe cases of COVID-19, accuracy was lower regarding milder cases. These findings suggest that the models perform well when diagnosing severe cases, but may require further improvements to accurately detect milder cases. This also highlights the difficulty confirming COVID-19 using other techniques such as polymerase chain reaction (PCR) testing, as well as potential bias stemming from false positives.

Second, when images of other pathologies similar to COVID-19 were included, this affected the model’s performance.

One of the major limitations of this study is the lack of metadata. Many of the currently available public databases contain no data on medical images. This drawback makes it difficult to convert current models into clinical applications. This research aimed to generate a database sufficiently representative of positive and negative COVID-19 cases. However, determining the variety of cases needs additional data such as age, sex, subject positioning, severity of the disease or contained pathologies.

## Conclusion

We presented a domain adaptation study and we applied it in the context of COVID-19 detection using chest X-ray images. The study used 26,047 images from 6 different data sources to fine-tune 3 pre-trained networks: IRV2, ResNet50, and DenseNet121. For the internal validation of the model, 2676 images from the 6 different data sources in training were employed. External validation of the models used 4098 images from 4 different sources.

Evaluation of the models revealed promising results in the internal validation set, showcasing accuracies ranging from 87 to 95%. However, these performance levels witnessed a significant decline when applied to the external dataset, with accuracies ranging from 61 to 78%. This contrast underscores the critical importance of assessing machine learning models across diverse datasets to guarantee that their performance is both robust and generalizable.

To improve the individual performance of the models, results from the 3 networks were combined by taking the weighted average of the output of the nodes, taking into account their entropy. This resulted in a balanced network that can detect both positive and negative cases with an accuracy of 81.16%, sensitivity of 80.97%, and specificity of 81.31% on external datasets. It is worth noting that these results present an important step forward toward utilizing a computer-based solution, with near real-time capabilities, compared to the time-intensive assessments carried out by expert clinicians.

Future research should include more models and investigate other methods for weighting networks aimed at more precise results in the detection of COVID-19 as well as apply to other domains. Additionally, deeper analysis leveraging metadata could provide insights into the limitations of the current study. These considerations contribute to a comprehensive understanding of the model’s applicability and potential refinements for broader applications across various domains.

## Data Availability

All data analysed during this study is included in this published article.
